# Non-suicidal self-injury, youth, and the Internet: What mental health professionals need to know

**DOI:** 10.1186/1753-2000-6-13

**Published:** 2012-03-30

**Authors:** Stephen P Lewis, Nancy L Heath, Natalie J Michal, Jamie M Duggan

**Affiliations:** 1 Department of Psychology, University of Guelph, Guelph, ON N1G 2W1, Canada; 2 Department of Educational and Counselling Psychology, McGill University, Montreal, QC H3A 1Y2, Canada

**Keywords:** Non-suicidal self-injury, Youth, Internet, Online activity, E-communities, Risks, Triggers, Monitoring, Assessment, Intervention

## Abstract

Non-suicidal self-injury (NSSI) content and related e-communication have proliferated on the Internet in recent years. Research indicates that many youth who self-injure go online to connect with others who self-injure and view others’ NSSI experiences and share their own through text and videos platforms. Although there are benefits to this behaviour in terms of receiving peer support, these activities can introduce these young people to risks, such as NSSI reinforcement through the sharing of stories and strategies, as well as, risks for triggering of NSSI urges. Due to the nature of these risks mental health professionals need to know about these risks and how to effectively assess adolescents’ online activity in order to adequately monitor the effects of the purported benefits and risks associated with NSSI content. This article offers research informed clinical guidelines for the assessment, intervention, and monitoring of online NSSI activities. To help bridge the gap between youth culture and mental health culture, these essentials include descriptions of Community, Social Networking, and Video/Photo Sharing websites and the terms associated with these websites. Assessment of these behaviours can be facilitated by a basic Functional Assessment approach that is further informed using specific recommended online questions tailored to NSSI online and an assessment of the frequency, duration, and time of day of the online activities. Intervention in this area should initially assess readiness for change and use motivational interviewing to encourage substitution of healthier online activities for the activities that may currently foster harm.

## 

Increasing attention is being paid to the nature of Internet use among youth [1], including reasons for these activities [2,3], and the risks with which they associate [e.g., [4]‐[7]. Recent efforts have focused on understanding the nature of non-suicidal self-injury (NSSI) activity and material on the Internet [8]‐[12]. This includes youth sharing their NSSI experiences through personal websites [9,11], communication amongst those who self-injure in virtual NSSI communities (e.g., message forums) [12] and major social networks (e.g., Facebook) [8], and sharing images and videos through popular e-platforms such as YouTube [7,8,10]. As noted below, findings suggest that there may be benefits, but also several risks associated with some forms of online NSSI content and communication. Mental health professionals working with youth engaging in NSSI need to be aware of these risks.

The objectives of the present paper are to (a) provide a brief review of the current literature examining the nature and scope of NSSI material on the Internet and its potential effect on those accessing the material, and (b) provide clinical guidelines for assessing, intervening, and monitoring a young person’s online NSSI activity. To identify papers, we used the terms: “self-injury,” “self-harm,” “online,” and “Internet,” in Psych-Info, PubMed and Google Scholar. Doing so yielded less than 20 studies, many of which were exclusively qualitative studies (e.g., discourse analysis) and which did not explicitly focus on risks of Internet activity related to NSSI (e.g., discussed how individuals used language to describe NSSI). Only papers explicitly examining or reporting potential risks and benefits associated with online NSSI activity were retained (n = 7); this number is indicative of the early state of this emerging field and highlights the critical need for further empirical investigation in the area. When relevant, we also included unpublished research (e.g., manuscripts under review, peer-reviewed conference presentations).

## Non-suicidal self-injury in youth

Non-suicidal self-injury (NSSI) is the direct, deliberate destruction of one’s own body (e.g., cutting, hitting or burning of the skin) in the absence of suicidal intent [13]. NSSI represents a salient mental health issue in adolescents with consistent lifetime prevalence rates of 13.9 to 21.4% [13,14] and an average of 13 incidents of NSSI occurring in the most recent 12-months among those who have self-injured [15]. NSSI may confer risk for suicide [16,17] and has numerous concomitants and risks, including: repeated NSSI, physical injury/scarring, dysregulated emotion, and various psychiatric symptoms (e.g., anxiety, depression) [13].

## Non-suicidal self-injury, youth, and the internet

Youth and young adults have daily Internet access and engage in more online social networking and video sharing than any other age group [1,18,19]. Since these age groups also have the highest NSSI rates [20], and adolescents who self-injure may engage in more online activity than those who do not self-injure [5,21], it is perhaps not surprising that there has been an influx of NSSI content online in recent years. In 2010, the International Society for the Study of Self-injury (ISSS) recognized the emergence of NSSI activity on the Internet and the importance of research in this area. Online communication about the impact of NSSI e-material has also received media attention. In 2011 alone, there were over 400 news stories published globally, most of which focused on the impact this e-material may have on those who access it [22].

Research indicates that the Internet may represent a preferred medium for otherwise isolated youth and young adults to communicate with others—namely, others who self-injure [9]‐[12,23,24]. One part of the Internet’s appeal stems from the anonymous nature of interaction it provides; indeed, research indicates that anonymous e-communication may hold particular appeal for those who experience psychological distress and other emotional difficulties [9,12,25,26] – many of the factors associated with NSSI risk [27].

## Benefits of online NSSI activity

The most commonly reported benefit associated with some online NSSI activity is that of social/peer support. Many youth and young adults who self-injure go online to share their NSSI experiences and connect with others who self-injure [10]‐[12,28]; this may be particularly appealing to individuals who may not feel comfortable discussing their NSSI experiences offline [9,10,12]. Furthermore, some research suggests that individuals involved in NSSI e-communities report reductions in NSSI behaviour subsequent to joining these groups [28]. In sum, there seem to be some advantages associated with some forms of online NSSI communication. To this end, more research is needed to determine the extent to which online NSSI activity has benefits and for whom. It will also be important to ascertain what types of online activities have benefits as well as the nature of these benefits (e.g., the impact of social support on NSSI behaviour). Although there may be some benefits associated with online NSSI activity, as discussed next, online NSSI activity may pose several risks.

## Risks of online NSSI activity

### Shared NSSI experiences & NSSI reinforcement

As noted above, a substantial number of individuals share their NSSI experiences with others through personal websites, discussion boards, general e-communities (e.g., question-and-answer websites), and video-sharing websites [7]‐[10,12,23,24,28,29]. Researchers have suggested that the manner by which some individuals share their NSSI experiences online may lead to reinforcement of the behaviour for some individuals when this e-material is repeatedly accessed [7,9,10,12,29,30]. This may also occur through virtual communication among those who self-injure. Indeed, bidirectional websites (e.g. discussion forums, video-sharing websites) permit users to not only access NSSI content, but to interact with other users about NSSI [7,10,12,29,30].

Many NSSI experiences disclosed online contain detailed descriptions of NSSI that emphasize emotional pain and suffering without a recovery-oriented message about prognosis [9,10]. Oftentimes, NSSI is presented as an effective means to cope with distress [7,9]. In some cases, NSSI is discussed as not always painful and/or as an unstoppable addiction [9,31]. In other cases, NSSI may be justified [7,9] or even glamourized [7,9,10]. Messages indicating that NSSI is not always painful and that little can be done to end NSSI may reinforce NSSI behaviour by virtue of impeding the likelihood of help seeking.

In addition to sharing NSSI experiences via personal websites and message boards, many individuals share NSSI videos online. In a descriptive study examining the content of NSSI videos collected at one time point in December, 2009 on YouTube [10], researchers examined 50 videos with a live person (i.e., character videos) and 50 with no live person (i.e., non-character videos). Collectively, these 100 videos represented the most viewed NSSI videos on YouTube (at the time of the study), with a total view count exceeding two million. These videos were favourably rated, as indicated by ratings from the community of users who watched them. The majority of NSSI videos had what the researchers referred to as “informational” (i.e., presented NSSI facts) and/or “melancholic/hopeless” (i.e., emphasized emotional pain) messages. Almost all non-character videos presented graphic NSSI photography, and 14 (of the 50) character videos depicted *in-action* NSSI. With over 5,000 total videos at the time of the study, “melancholic/hopeless” videos about NSSI that present graphic imagery may not only be frequently viewed, but widely accessible. Similar to text-based NSSI websites, content in online NSSI videos may reinforce NSSI if they are repeatedly viewed by reinforcing the notion that NSSI is a viable response to distress and one that is difficult to overcome.

In a follow-up study, the content of viewers’ comments to these NSSI videos were examined as an index of viewer response [30]. Most responses consisted of viewers sharing their own NSSI experiences; many responses also validated and/or praised uploaders for their videos. Few discussed or mentioned NSSI recovery; most comments indicated that the individual was still injuring. Taken together, multiple messages discussing NSSI experiences without an emphasis on recovery may reinforce NSSI for those who access these comments. Moreover, convergent responses offering praise and validation to videos that are “melancholic/hopeless” and that contain graphic NSSI imagery may further reinforce NSSI – for uploaders as well as those who read these comments and view these videos.

The extent to which NSSI is reinforced through online NSSI activity merits research attention; indeed, this is a critical issue to address, as outlined by several researchers in this growing field [7,9,10,12]. Research in this area should investigate the nature of the relation between online activity related to NSSI and NSSI thoughts and behaviour. In particular, it will be important to examine whether different online activities (e.g., accessing material, uploading material), and materials (e.g., text, imagery) associate with continued NSSI and perceptions of recovery (e.g., viewing recovery as possible, wanting to recover).

## Shared NSSI strategies & reinforcement

On many websites and e-forums, NSSI methods and tips about how to conceal the behavior are shared between users [9,12]. First-aid tips are also shared, including ways to prepare oneself for NSSI (e.g., cleaning a razor) and how to tend to wounds after NSSI (e.g., how to clean a wound) [9]. Adolescents who self-injure and who access this material may therefore be exposed to learning new ways to self-injure, how to prepare for and carry out NSSI, and how to hide this from others (e.g., friends, family). This may thwart the likelihood of help seeking for some individuals. It may also engender the belief that help is not needed for NSSI. Thus, in addition to youth learning new NSSI-related strategies the nature of this content may reinforce NSSI. Similar to the above noted need to examine the phenomenon of NSSI reinforcement, the impact of sharing NSSI strategies should also be studied further. For example, it will be important to understand whether some youth are more influenced by these strategies and whether this associates with aspects of NSSI behaviour (e.g., using new methods to injure).

## Triggering NSSI urges

Many websites and e-communities post trigger-warnings [9,10]. These warnings are intended to warn users that website content may trigger NSSI. That is, as a result of accessing NSSI content, individuals may experience emotional upset, and with that, an increased urge to injure; in turn, this may lead to NSSI engagement. Recent findings provide initial support for this. In a content analysis of personal NSSI websites, several individuals reported on their website that they experienced NSSI urges and even self-injured pursuant to seeing NSSI imagery or reading graphic NSSI descriptions [9]. In another study examining users’ responses to NSSI photographs shared within an e-forum, some individuals reported that seeing NSSI images triggered them and/or would trigger others to self-injure whereas others reported that the images were not triggering [32]. Thus, although online activities as a whole have not been proven to result in self-injury for all viewers, collectively, these findings provide preliminary support for the widespread clinical assumption that some people are triggered by graphic NSSI material. Moreover, these findings indicate the need to further explore how people may be differentially impacted by NSSI images (e.g., who is impacted, how people are impacted).

In summary, it is likely that many individuals who self-injure also engage in online activities related to NSSI. Despite the mentioned benefits associated with these online activities (e.g., support from others, reports of reduction of NSSI), there are also potential risks (e.g., maintenance of the behavior, triggering material). Therefore, it is important for clinicians who work with adolescents who self-injure to consider online activity in assessment and treatment contexts, at least to assess the possible benefit or harm for the particular client – a sentiment echoed in calls from researchers to assess Internet activities of youth who self-injure in clinical contexts [7]‐[9,12,29]. Although some general guidance strategies have been offered [8,29], to date, there is a lack of detailed guidelines pertaining to how Internet activities could be assessed and monitored. An assessment rubric is provided below for professionals working with youth who engage in NSSI concerning their online activities. Of course, the focus on assessing, monitoring and intervening around online activities is done within the broader context of the overall functioning of the youth. It is important to acknowledge that due to the limited research in the area, these guidelines are tentative but empirically informed and derived from research examining approaches used to manage NSSI [27,33] and problematic online behaviour among youth [34].

## Orientation for clinical work

When working with youth who engage in NSSI, the scope and nature of the patient’s online activities must be addressed. However, the precise manner of how to comprehensively assess such online activities is rarely addressed within the literature. Below, the essentials of assessment, intervention, and monitoring are described.

First, in order to effectively interact with youth regarding their online NSSI activities it is important to have a general awareness and understanding of the nature of possible online activities, including general knowledge of the language typically used by youth when describing these activities. Although the majority of youth are extremely knowledgeable about the diversity of potential online activities, some clinicians may be less aware [29]; this may make assessing and monitoring these activities very difficult. These activities fall within the broad categories of Community, Social Networking, and Video/Photo Sharing websites. It is important to acknowledge that these activities may overlap; for example, community websites may also have space for photo sharing. Furthermore, it is essential for clinicians to have knowledge of the language specific to these activities in order to have credibility with youth. Table1 provides descriptions of the different types of activities within each website category (including example websites), with suggested assessment questions associated with these activities. These questions tap into important information and provide clinicians with examples of appropriate, contemporary language for assessing online activities. Clinician familiarization and exploration of activities and websites is recommended.

**Table 1:** Internet Factsheet: NSSI Related Activities

	**Community Websites**	**Social Networking Websites**	**Video/Photo Sharing Websites**
**Terms**	Chat Forum: Space dedicated to real time chat among individuals who are accessing the website. Moderated: Content and membership on website is controlled and regulated by creator. Discussion Forum (message board): online space where users can openly exchange information and opinions regarding a common interest/theme. e-communities: electronic community/social network of users who share a common interest. Peer driven: Created and moderated by a non-professional. Professionally driven: Created and moderated by a mental health professional.	▪ **Facebook:** Friends: People you connect and share profile information with. Post: Public sharing of information on a wall. Profile: User space containing personal information, online exchanges and photos. Wall: User profile space where friends can post and share information. ▪ **MySpace**: Blog: A personal journal created by user. ▪ **Twitter**: Followers: size of audience following individual’s tweets/profile. Tweet: real time information sharing in 140 characters or less. ▪ **General (common terms found across all social networking websites).** Group: collection of individuals who keep in touch surrounding a particular theme. Instant chat/messaging (IM): Live, real time chat that occurs in present time between members. Members: individuals who join a group Messages: Private exchange of material (e.g., messages, photos). Public vs. Private group: Membership required.	Account: viewer to verify they are a mature audience (18 years and older). Character: Videos containing live individual(s) Comments: Public remarks/observations posted by video viewers pertaining to a specific video. Non-Character: Videos containing visual representations such as images, video stills, and/or text. Subscribe: To receive updates when a specific video uploader posts new videos. Top Favorited: A user indicates a specific video is their preferred. Video Uploader: User who creates and shares videos. Video view count: Number of video views, also referred to as “hits”.
**Examples**	http://self-injury.net* http://www.psyke.org/*	http://www.facebook.com http://www.myspace.com http://www.twitter.com (account needed to access groups)	http://www.youtube.com http://www.flickr.com

*Note*: * websites are examples and are not suggested as recommendations.

A second consideration when working with youth around their online NSSI activities is the pervasiveness of online activities in their lives and the discrepancy in perspectives regarding online activities between adolescent culture and that of many mental health professionals. Online activities are an intrinsic part of adolescents’ lives and the use of e-communities and social networking sites for social support concerning any issue is commonplace, whereas for many mental health professionals the pervasiveness of online activities in the lives of their patients is not necessarily relevant, understandable, or recommended. Indeed, for a large number of youth, online interactions are (a) an inherent part of their culture, (b) highly accessible and available, and (c) often very enjoyable. Because online activities are so accepted, pervasive, and rewarding it may be very challenging to stop them - even when assessment suggests they may be harmful. Thus, the assessment, intervention, and monitoring of this behaviour is complex and cannot be avoided by simply asking the patient to stop all online activities.

## Assessment

Assessment of NSSI online activities should follow a basic Functional Assessment approach. Here, youth should be asked to keep their own weekly log (i.e., between sessions) to record all of their online activities. Specifically, the youth should record: (a) events/interactions, thoughts, and feelings that *preceded the online activity*, (b) the events/interactions online and the thoughts and feelings *during the online activity*, and the (c) events/interactions, thoughts, and feelings *following the online activity.* During the next session a broad assessment of online activities can be conducted using the Recommended Questions about Online Activity Section I-Activity Type (Table2), to explore the type of activities enacted. Completing the broad assessment of activities will establish the types of activities, interactions, and material accessed by the adolescent. This may reveal obvious potential triggers for harm, or may suggest a source of support for the youth regarding their recovery attempts. It is important, however, to not limit the assessment to the exploration of activity-type as adolescents may not accurately report, or be aware of, the effects of their online activities on thoughts and mood.

**Table 2:** Recommended Questions about Online Activity

**I. Activity Type**			
	*Review log:* What type(s) of activities do you engage in online activities, concerning NSSI (Informational, interactive, social networking, and video viewing/sharing/posting)?		
	**Community**		
		What are the resources available?	
		Is this website professionally or peer driven? Moderated?	
		What specific activities do you engage in on these websites (live chat, posting, information seeking)?	
	**Social Networking**		
		What social networking websites are you affiliated with?	
		Do you have friendships/connections with people online surrounding NSSI?	
			*If yes,* what is the nature of the relationship(s)
			*If yes,* what is the nature of the relationship(s)
		Are you a member of any group related to NSSI?	
			*If yes,* what are the themes surrounding that group (against NSSI, pro NSSI, neutral)?
			*If yes,* is this group public or private?
			*If yes,* is it moderated?
		Are there any visual representations of NSSI among these groups?	
		What specific activities do you engage in on these websites (live chat, messaging, posting, information seeking)	
	**Video/Picture Sharing**		
		What specific websites do you visit?	
		Do you create videos/photos related to NSSI?	
			*If yes,* discuss themes/content of videos created.
			*If yes,* are these videos character or non-character videos?
			*If yes,* what purpose does creating these videos serve (creative outlet)?
		What types of videos/photos do you watch?	
		Are these character or non-character videos?	
		What are the general themes in these videos (against NSSI, pro NSSI, neutral)?	
		Do these videos present visual presentations of NSSI?	
			*If yes,* are these visual presentations accompanied by a warning?
		Are these visual presentations of NSSI triggering?	
			*If yes,* discuss nature, intensity and degree of triggering material.
		What other specific activities do you engage in on these websites (messaging, commenting, following channels)?	
**II. Frequency**			
	*Review log:* Discuss frequency of NSSI online activities (explore usage, during week and weekend).		
**III. Functional Assessment of NSSI behaviours in relation to Internet activities**			
	*Review log:* When/why did you first start engaging in NSSI online activities? Explore first episode.		
		Has your self-injury increased/decreased/remained the same since you began engaging in NSSI online activities?	
		What are events/interactions, thoughts, and feelings that preceded/occur during/follow the online activity?	
		Do you self-injure before/after engaging in NSSI online activities?	
			*If yes,* explore online activities that may confer/reduce NSSI risk.

Next, a brief assessment of the frequency, duration, and time of day of online activities (cumulatively, including all activities) fosters awareness of the extent of the exposure. If it becomes clear online behaviours are disrupting daily life activities (e.g., school, sleep, eating), this is an added concern. Finally, a review of the functional log is essential to understand the function of the online activities and to effectively evaluate the potential harm/benefits of the behaviour to the youth. Evaluating possible antecedents contributing to the youth seeking out unhealthy online activities is useful when reviewing the function log, however, a natural limitation of having a client use a functional assessment to record their online activities is the potential problems with self-report accuracy. While determination of potential harm is a central factor in the log analysis, it is important to recognize that, for some youth, online activities may provide much needed support. Clinicians need to be cautious in assuming that all online activities will pose harm. If the functional analysis reveals behaviours that are clearly impeding the youth from recovery then intervention to change the online activities is needed.

## Intervention

As indicated earlier, altering online activities in youth may be extremely challenging. Asking the youth to stop engaging in the online activities may result in the behavior becoming secretive, which compromises treatment. It is recommended that the first step in attempting to change the behavior is to assess the readiness for change using the stages of change model [35]. The stages of change approach has been used for the treatment of Internet addiction [e.g., [34,36] and may be useful in approaching youth who are deeply invested in online NSSI activity. A particularly helpful aspect of this approach is motivational interviewing, which enhances the youth’s desire to change his/her online activities. Although motivational interviewing has not been used directly to alter online NSSI activities, Kress and Hoffman [33] have used it to increase motivation to change amongst individuals who self-injure; it has also been used to increase motivation to change among individuals with Internet addiction [34].

In seeking to effect change in online NSSI activities of youth it is suggested that beyond encouraging offline activities, substituting healthier online activities may be more effective than attempting to eliminate online activities altogether. Redirecting youth to healthier online activities with continued monitoring of the effects of these activities through the log review is recommended with the goal of establishing a pattern of online behaviours that is beneficial (and not harmful/triggering) to the youth. From here, each session should include a brief probe concerning online activities, which can be integrated as a part of the overall assessment of the youth’s functioning. Sudden changes in online activities may signal a change in the adolescent’s emotional wellbeing.

Establishing a healthy online behaviour repertoire is significantly aided by providing a list of recommended NSSI websites. Recently, in response to their research examining online NSSI activity by adolescents and young adults, two of the authors developed an outreach website to address concerns about the risks associated with online NSSI activity. This website, Self-injury Outreach and Support, provides empirically based NSSI information and helpful recovery-focused resources to those who self-injure, those who have recovered, caregivers and families, friends, teachers and the health professionals who work with individuals who self-injure. Other websites also provide excellent NSSI resources. Table3 outlines these websites. As online activities change and new websites emerge, it is suggested that clinicians develop a list of recommended activities that are regularly updated for distribution to clients.

**Table 3:** Websites providing NSSI and mental health resources

	**Recommended Activities**	**Resources Available**
**Community Websites**	▪ **Self Injury Outreach & Support** SIOS http://sioutreach.org ▪ Highlights: Information for those who self-injure, parents, teachers, peers, partners, mental health and medical professionals, sharing of NSSI recovery stories, various NSSI resources. ▪ **Cornell Research Program on Self-Injurious Behaviour** CRPSIB http://www.crpsib.com/ Highlights: Research publications, resources, factsheets, video presentations on treatment ▪ **Self Abuse Final Ends (SAFE)SAFE Alternatives** http://www.selfinjury.com/ Highlights: Admission, treatment and referral information, resources, moderated blog, materials for mental health professionals. **▪ Self Injury Foundation** SIF http://www.selfinjuryfoundation.org/ Highlights: Up to date news about self-injury, volunteering possibilities, moderated blog, resources, research publications.	**▪** Professionally driven websites that offer information and credible resources concerning self-injury. **▪** All material is moderated and websites are void of triggering material. **▪** Psychoeducational material, support, and resources available for individuals who currently self-injure and who have recovered, as well as their friends and family. **▪** Coping resources for individuals who self-injure **▪** Resources (i.e., factsheets and research articles) also available for mental health professionals. **▪** Websites also promote awareness and advocacy regarding self-injury.
**Social Networking Websites**	**▪** Activities and specific videos are not recommended	**▪** The nature of social networking websites does not guarantee that uploaded/shared content (i.e., discussions, real time chat exchanges, photos, videos) is moderated; therefore some material may be triggering and/or NSSI-reinforcing.
**Video/Picture Sharing Websites**	**▪** Activities and specific videos are not recommended.	**▪** The nature of video and photo sharing websites exposure users to a wide array of NSSI videos that may vary in content. The nature of video/photo sharing websites do no guarantee that posted videos and photos are moderated, and they may contain NSSI imagery and content that may serve be triggering and/or NSSI-reinforcing. ▪ Comments and public exchanges regarding posted videos/photos are also typically not moderated and may also contribute to NSSI reinforcement
**General Mental Health Websites**	**▪** Reach out* http://au.reachout.com// Highlights: An Anonymous help-line website for youth. Contains diverse information, stories, videos, blogs, and forums regarding many issues including mental health difficulties, self-injury, alcohol and drugs, family and relationships, independence, loss and grief, physical health issues, safety and violence, school, sex and pregnancy, and sexuality. **▪** Mind Your Mind* http://mindyourmind.ca/ Highlights: Designed specifically for youth and young adults regarding general mental health issues. Treatment/outreach information, personal stories, videos, music, discussion boards, an online community, coping tools and interviews. **▪** Teen Central.Net* http://www.teencentral.net Highlights: Anonymous help-line website for youth regarding physical and mental health issues. Safe sharing is ensured by being professionally created and moderated, and an account is required by all members. Personal stories, links to teen help-line, and youth friendly health care videos. Parental resources also available. **▪** Mayo Clinic http://www.mayoclinic.com/ Highlights: An online health community designed to provide health information about a number of diseases and conditions including a definition, symptoms, causes, risk factors, complications, preparing for your doctor appointment, tests and diagnosis, treatments and drugs, lifestyle and home remedies, coping and support, and prevention. **▪** Teen Mental Health http://teenmentalhealth.org/ Highlights: Comprehensive information available for health professionals, school mental health educators, families and youth regarding mental health issues. Content includes psychoeducational materials, video presentations (virtual classrooms) and personal story sharing.	▪ Professionally driven websites that offer information and credible resources concerning general mental health issues. ▪ All material is moderated and websites are void of triggering material. ▪ Psychoeducational material, support, and resources are available for youth and young adults with mental health issues, as well as their family, educators, mental health professionals, and friends. ▪ *Material is presented specifically in a youth appropriate and friendly manner. ▪ Outreach and treatment resources are presented for individuals with mental health issues.

## Summary

Research conducted over the past several years has helped to advance knowledge about the nature of NSSI content and communication on the Internet. Although there may be some benefits associated with some online NSSI activity, there are a number of risks meriting the attention of mental health professionals who work with adolescents who self-injure. When working with youth who engage in both NSSI and related online behaviour, it is important to conduct a comprehensive assessment to gain insight into the types and extent of activities and their corresponding antecedents and consequences. A Functional Assessment can help determine what may be reinforcing about the youths’ online activity and how this may impact NSSI. From here, approaches rooted in the stages of change model [35] and motivational interviewing [33,35,36] may be particularly useful when monitoring these activities, reducing the impact of more pernicious online material, and helping adolescents who self-injure develop healthier online behaviours.

## Competing interests

The authors declare that they have no competing interests.

## Authors’ contributions

SPL, NLH, NJM and JMD conceptualized the overall organization of the manuscript. SPL wrote the first section and NLH wrote the second. SPL, NLH, NJM and JMD edited the entire manuscript. NJM & JMD developed the tables. All authors read and approved the final manuscript.

## References

[B1] Rideout V Foehr UG Roberts DF Generation M2: Media in the Lives of 8- to 18-Year-Olds 2010 Menlo Park: Kaiser Family Foundation

[B2] Lenhart A Madden M Social networking websites and teens: An overview 2007 Washington, DC: Pew Internet & American Life Project

[B3] Lenhart A Teens and Social Media: An Overview 2009 Washington, DC: Pew Internet and American Life

[B4] Borzekowski D Schenk S Wilson J Peebles R e-Ana and e-Mia: A content analysis of pro-eating disorder Web sites Am J Public Health 2010 100 Suppl 8 1526 1534 20558807 10.2105/AJPH.2009.172700 PMC2901299

[B5] Mitchell K Ybarra M Online behavior of youth who engage in self-harm provides clues for preventive intervention Preventive Medicine: An International Journal Devoted To Practice And Theory 2007 45 Suppl 5 392 396 10.1016/j.ypmed.2007.05.008 17599399

[B6] Shaffer H Hall M Vander Bilt J 'Computer addiction': A critical consideration Am J Orthopsychiat 2000 70 Suppl 2 162 168 10826028 10.1037/h0087741

[B7] Whitlock J Purington A Gershkovich M Media, the internet, and nonsuicidal self-injury 2009 Washington, DC: American Psychological Association 139 155

[B8] Duggan JM Heath NL Lewis SP Baxter AL An examination of the scope and nature of non-suicidal self-injury online activities: Implications for school mental health professionals School Mental Healthin press

[B9] Lewis SP Baker TG The possible risks of self-injury web sites: a content analysis Arch Suicide Res 2011 15 Suppl 4 390 396 22023646 10.1080/13811118.2011.616154

[B10] Lewis SP Heath NL St Denis J Noble R The scope of nonsuicidal self-injury on YouTube Pediatrics 2011 127 Suppl 3 e552 e557 21339269 10.1542/peds.2010-2317

[B11] Rodham K Gavin J Miles M I hear, I listen and I care: a qualitative investigation into the function of a self-harm message board Suicide Life Threat Behav 2007 37 Suppl 4 422 430 17896882 10.1521/suli.2007.37.4.422

[B12] Whitlock JL Powers JL Eckenrode J The virtual cutting edge: The internet and adolescent self-injury Dev Psychol 2006 42 Suppl 3 407 417 16756433 10.1037/0012-1649.42.3.407

[B13] Nock MK Favazza AR Nonsuicidal self-injury: Definition and classification 2009 Washington, DC: American Psychological Association 9 18

[B14] Jacobson CM Gould M The epidemiology and phenomenology of non-suicidal self-injurious behavior among adolescents: A critical review of the literature Arch Suicide Res 2007 11 129 147 10.1080/13811110701247602 17453692

[B15] Lloyd-Richardson EE Perrine N Dierker L Kelley ML Characteristics and functions of non-suicidal self-injury in a community sample of adolescents Psychol Med 2007 37 Suppl 8 1,183 1,192 10.1017/S003329170700027X PMC2538378 17349105

[B16] Nock MK Joiner TE Gordon KH Lloyd-Richardson E Prinstein MJ Non-suicidal self-injury among adolescents: Diagnostic correlates and relation to suicide attempts Psychiatry Res 2006 144 Suppl 1 65 72 16887199 10.1016/j.psychres.2006.05.010

[B17] Glenn C Klonsky E Social context during non-suicidal self-injury indicates suicide risk Pers Indiv Differ 2009 46 Suppl 1 25 29

[B18] Lenhart A Madden M Hitlin P Teens and technology: Youth are leading the transition to a fully wired and mobile nation 2005 Washington, DC: Pew Internet and American Life

[B19] Lenhart A Madden M Macgill AR Smith A Lenhart A Madden M Macgill AR Smith A Teens and social media: The use of social media gains a greater foothold in teen life as they embrace the conversational nature of interactive online media 2007 Washington, DC: Pew Internet & American Life Project

[B20] Rodham K Hawton K Epidemiology and phenomenology of nonsuicidal self-injury 2009 Washington, DC: American Psychological Association 37 62

[B21] Heath NL Baxter AL Toste JR McLouth R Adolescents’ willingness to access school-based support for nonsuicidal self-injury Can J Sch Psychol 2010 25 Suppl 3 260 276

[B22] Sornberger MJ Heath NL Lewis SP The Digital Butterfly Effect: Knowledge Transfer and NSSI Research 2011 New York

[B23] Lewis SP Rosenrot S Messner M Davis M I have a question: What do people ask about self-injury online? 2011 New York

[B24] Lewis SP Rosenrot S Messner M Seeking validation in unlikely places: What people ask about non-suicidal self-injury online, under review 10.1080/13811118.2012.695274 22852787

[B25] Bargh JA McKenna KYA Fitzsimons GM Can you see the real me? activation and expression of the "true self" on the internet J Soc Iss 2002 58 Suppl 1 33 48

[B26] McKenna KYA Green AS Gleason MEJ Relationship formation on the internet: What's the big attraction? J Soc Iss 2002 58 Suppl 1 9 31

[B27] Klonsky ED Muehlenkamp JJ Lewis SP Walsh B Nonsuicidal self-injury 2011 Cambridge: Hogrefe Publishing 92 vi

[B28] Johnson GM Zastawny S Kulpa A E-message boards for those who self-injure: Implications for e-health Int J Ment Heal Addict 2010 8 Suppl 4 566 569

[B29] Whitlock J Lader W Conterio K The internet and self-injury: What psychotherapists should know J Clin Psychol 2007 63 Suppl 11 1135 1143 17932984 10.1002/jclp.20420

[B30] Lewis SP Heath NL Sornberger M Arbuthnott A Helpful or harmful? An examination of viewers' responses to non-suicidal self-injury videos on YouTube J Adol Healthin press 10.1016/j.jadohealth.2012.01.013 22999839

[B31] Lewis SP Rodham K Gavin J St Denis J Once you start, you can’t stop: Is self-injury an addiction? 2011 New York

[B32] Baker TG Lewis SP Attitudes toward Online Photographs of Non-suicidal Self-Injury: A Thematic Analysis under review 10.1080/13811118.2013.805642 23889572

[B33] Kress VE Hoffman RM Non-suicidal self-injury and motivational interviewing: Enhancing readiness for change J Ment Health Couns 2008 30 Suppl 4 311 329

[B34] Griffiths MD Meredith A Videogame addiction and its treatment J Contemp Psychother 2009 2009 39 247 253

[B35] Prochaska JO Velicer WF The transtheoretical model of health behavior change Am J Health Promot 1997 12 38 48 10.4278/0890-1171-12.1.38 10170434

[B36] Miller WR Rollnick S Motivational interviewing: Preparing people to change addictive behavior 1991 New York: Guildford Press

